# Structure and Mechanical Properties of the NiTi Wire Joined by Laser Welding

**DOI:** 10.3390/ma16072543

**Published:** 2023-03-23

**Authors:** Tomasz Goryczka, Karol Gryń, Adrian Barylski, Barbara Szaraniec

**Affiliations:** 1 Institute of Materials Engineering, University of Silesia in Katowice, 75 Pułku Piechoty 1A, 41-500 Chorzów, Poland; adrian.barylski@us.edu.pl; 2 Faculty of Materials Sciences and Ceramics, Department of Biomaterials and Composites, AGH University of Science and Technology, al. Mickiewicza 30, 30-059 Kraków, Poland; kgryn@agh.edu.pl (K.G.); szaran@agh.edu.pl (B.S.)

**Keywords:** NiTi shape memory alloy, welding, microhardness, pseudoelasticity

## Abstract

Joining wires made of NiTi alloys with shape memory effect and pseudoelasticity causes many technical and structural problems. They result from unwanted phase interactions that occur in high temperatures and negatively affect the characteristics of these materials. Such obstacles are challenging in terms of welding. Hence, an attempt was made to join NiTi wires via an economical and reliable basic laser welding technique which does not require complicated equipment and gas protection. The parameters such as spot diameter and pulse time were constant and only the laser power, calculated as a percentage of the total power, was optimized. The wires were parallelly connected with overlapping seam welds 10 mm long. The welds were examined regarding their microstructure, chemical and phase composition, reversible martensitic transformation, microhardness, and pseudoelasticity. The obtained results showed that the joint was completed at the 12–14% power. The weld revealed good quality with no voids or pores. As the laser power increased, the microhardness rose from 282 (for 4%) to 321 (for 14%). The joint withstood the stress-inducing reversible martensitic transformation. As the transformation was repeated cyclically, the stress value decreased from 587 MPa (initial wire) to 507 MPa (for the 14% power welded wire).

## 1. Introduction

Due to their shape memory phenomena, NiTi alloys are known for numerous practical applications [1]. Although almost 50 at.% of content is taken by nickel, the alloys reveal good corrosion resistance. Therefore, they are commonly used for medical implants as well as elements of medical instruments [2,3,4,5]. Modern medicine is consistently striving to use less-invasive treatments. For instance, implants are introduced with guide wires through veins, arteries, or urinary ducts which do not exceed a few millimeters in diameter [6,7]. Such procedures require the implant’s size and volume to match the available diameter of the supply canal. That is why it is essential to reduce the size of NiTi implant elements made of wires, rods, and tapes measuring below 1 mm in diameter. A good example are stents made of wires or flat bars whose forming elements range from several hundred micrometers to 0.8 mm [8,9,10].

In order to create a complex, three-dimensional structure of an implant, an appropriate method of forming and connecting elements or their fragments is necessary. There are several techniques of joining NiTi alloys, including: plasma welding [11], vacuum brazing [12], resistance welding [13], tungsten inert gas [14], friction welding [15,16], microplasma arc welding [15], capacitor discharge welding [15], explosive welding [16], ultrasonic welding [16], adhesives joining [16], and laser welding [15,16,17,18,19,20,21]. However, most of these methods are used to join sheets, tubes, tapes, or strips. For thin wires, the laser welding is an efficient, economical, and uncomplicated technique that can join particularly thin wires applied in medical procedures [20,22]. Thanks to this method, one can control the size of the welded area on a micro-scale. Moreover, it is unnecessary to use fluxes, solvents, or other additives. Therefore, the material is free from cytotoxic substances. In addition, at high temperature locally generated by the laser beam, the welding process supports the material’s sterilization.

Not much literature is devoted to joining thin wires made of NiTi alloy of less than 0.5 mm in diameter. The papers reported the possibility of butt welding of NiTi wires of 100 µm whose strain was 3% at a tension of 500 MPa [17]. In work [20], the welding parameters for a 0.5 mm wire were optimized by selecting the power range of 54 W–72 W. The welding time was relatively long (85–115 ms), but the authors did not analyze the influence of welding conditions on pseudoelasticity. Chan et al. [23] showed the cycling repetition of pseudoelasticity for butt welded 0.5 mm wires whose maximum strain was 4%. Another method of joining wires with a stitch parallel to the wire axis was proposed in [19]. Two NiTi wires of 0.44 mm, intended for cardiac stents, were connected parallelly. The microstructure, thermal transformation behavior, and the strength of the obtained weld were analyzed, yet, again, no attempt was made to assess their pseudoelasticity.

Therefore, in the presented work, we supplemented the reported results with the study of cyclically generated pseudoelasticity in the parallelly connected thin NiTi wires. We also revealed how the laser welding parameters, performed with a low-power device, influenced the weld microstructure, related to the thermal reversibility of martensitic transformation and microhardness. The tests were conducted on the elements made of 0.4 mm wires intended for cardiac surgeries.

## 2. Materials and Methods

**Figure 1:**
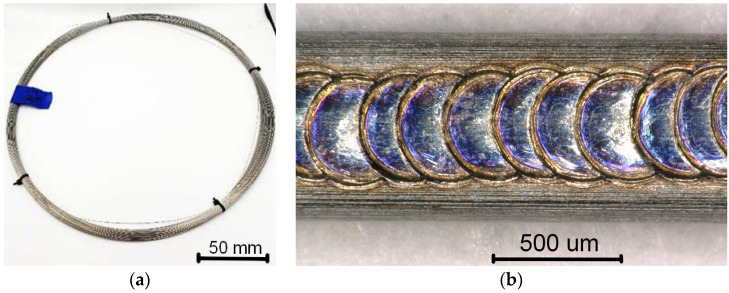
As-delivered NiTi wire (**a**) and an example of the overlapping seam weld (**b**).

The studies were carried out on a commercially available NiTi wire with a diameter of 0.4 mm (Figure 1a). Two 45 mm long wires were bonded parallelly with the 10 mm overlapping seam weld (Figure 1b). The welding was performed on a desktop jewelry welding machine (PixoLaser OPT-JW100, Shenzhen City, China). In order to investigate the influence of laser welding on the material’s characteristics, only one parameter was customized—the power of the laser beam (Curr%). For convenience, it was calculated as a percentage of the total laser power (100 W). The Curr equal to 4, 8, 10, 12, and 14% was applied to obtain the overlapping seam weld. The other welding parameters were constant: the laser beam’s cross-section was circular with the 0.1 mm spot diameter and the 0.4 ms pulse duration.

The welds’ microstructure was observed on their cross-sections using the digital microscope VHX-900F (KEYENCE, Osaka, Japan) and the scanning electron microscope (SEM) JSM 4680 (JEOL, Tokyo, Japan). The SEM was operated at 20 kV and equipped with an X-ray energy dispersive spectrometer (EDS). The 3 mm long samples were embedded in graphite and polished with sandpaper. The final polishing was carried out with polishing pastes of 1µm gradation. The surface was etched in a H_2_O:HNO_3_:HF solution in the 10:5:1 ratio.

The thermal behavior of the martensitic transformation was studied using the Mettler Toledo DSC 1 (Greifensee, Switzerland) differential scanning calorimeter (DSC).

The structural examination was performed via X-ray diffraction patterns measured with the X’Pert-PRO diffractometer (PANalytical, Almelo, the Netherlands) with CuKα_(1 and 2)_ radiation. The measurements were taken at room temperature using the Bragg–Brentano geometry at the step-scan mode in an angular 2θ range: 10–140°. The ICCD-PDF4 (International Centre for Diffraction Data) database was used for phase identification.

The micromechanical tests were performed with the Micro Combi Tester—MCT3 device (Anton Paar, Corcelles-Cormondrèche, Switzerland). The measurements complied with the ISO 14577 standard. The Berkovich diamond indenter (B-V 83) was used with the maximum load of 250 mN and a load/time of 30 s. The HV_IT_ hardness (hardness determined during indentation) was determined by the Oliver–Pharr method [24,25].

**Figure 2:**
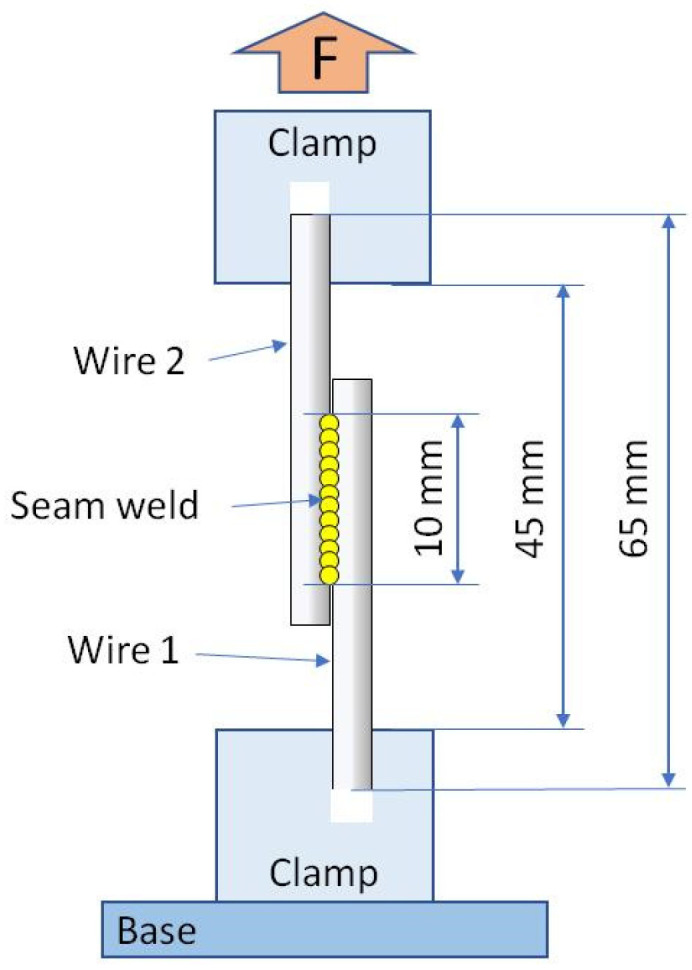
Scheme of mounting the welded wires in the tensile test holders.

The tensile test to determine the shear strength of the weld was performed on the universal testing machine Zwick 1435 Retroline (Zwick Roell, Ulm, Germany) equipped with the 5 kN load cell. The measurements were carried out at room temperature, following the ASTM F2516 standard due to the practical application of welded wires. The diagram of the sample mounting and the acting force is shown in Figure 2.

## 3. Results and Discussion

### 3.1. The Initial State of the Wire

**Figure 3:**
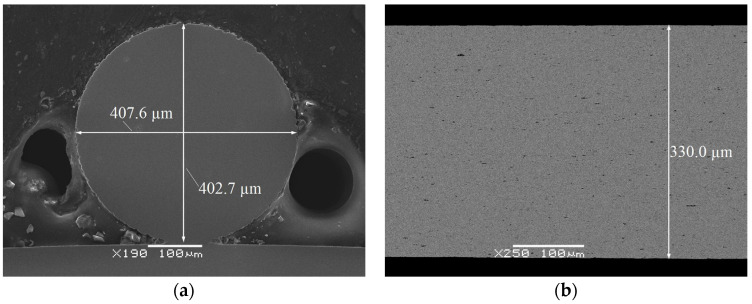
Images of the wire’s cross-sections: (**a**) transverse (SEM-SE); (**b**) longitudinal (SEM-BS).

**Figure 4:**
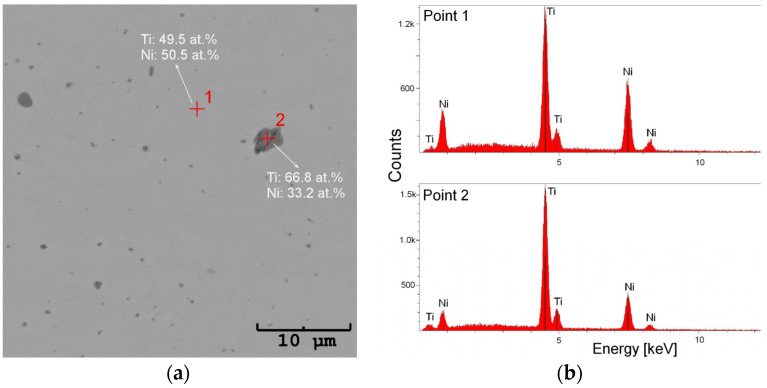
SEM image of the transverse wire’s cross-sections with Ti_2_Ni particles (**a**) as well as the measured EDS spectra (**b**).

In order to characterize the wire before welding, the detailed analyses of the microstructure and chemical composition were carried out on transverse and longitudinal cross-sections. An example of microscopic observations is shown in Figure 3. The images revealed the compact structure of the wire, free of discontinuities, pores, or micro/macro cracks. However, there were numerous particles ranging from several tens of nanometers to several micrometers in size (Figure 3). The average chemical composition of the matrix oscillated around the equiatomic one. A slight nickel predominance with the differences in tenths of a percent was found, practically the same in both cross-sections (Table 1). The chemical composition analysis of the particles showed that they contained almost twice as much titanium as nickel—which is characteristic of the Ti_2_Ni equilibrium phase (Figure 4).

**Figure 5:**
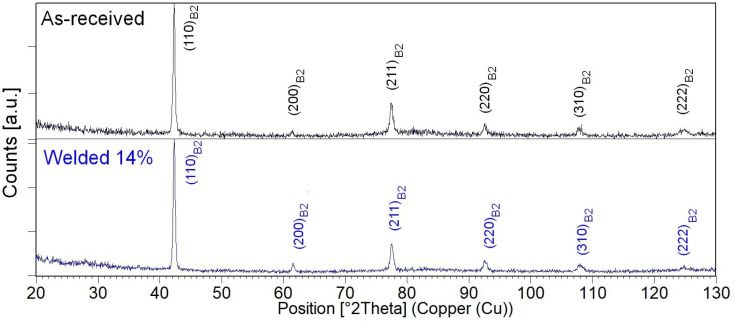
X-ray diffraction patterns measured for the as-received (black-line) and the welded wires with the Curr power of 14% (blue-line).

The phase analysis of the measured diffraction pattern showed that the matrix consisted of the β-NiTi intermetallic phase. Depending on the temperature, this phase can reveal a high-temperature B2-type or a low-temperature structure with the B19′ monoclinic martensite. In our study, the wire’s distribution of diffraction peaks and their intensity corresponded only to the B2 structure (ICDD card no. 65-0917) (Figure 5). The parent phase present at room temperature met the requirements for pseudoelasticity [26].

### 3.2. Weld Characteristics

**Figure 6:**
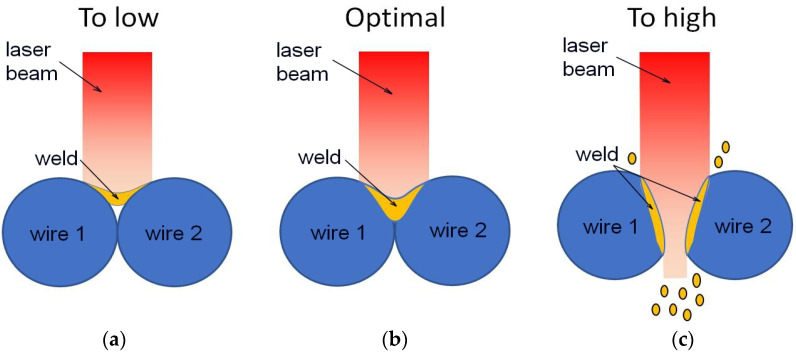
Scheme of the formation of the weld cross-section depending on the applied laser power.

The applied welding parameters influenced the weld quality, shape, structure, phase, and chemical composition—thus the mechanical properties. Depending on the laser power, the weld is formed in the joined material as a one of the three types (Figure 6). The insufficient laser power melted a too-small amount of the material and the weld did not sufficiently fill the area between the connected wires (Figure 6a). On the contrary, too high power led to the perforation of the connected wires and the weld itself (Figure 6c). The optimally selected power formed the complete weld between the joined wires with the cross-sectional shape shown in Figure 6b.

Due to the form of the welded material—a wire of 0.4 mm in diameter,—the parameters such as pulse time and spot size were set as constants of 0.4 ms and 0.1 mm, respectively. The laser power calculated as a percentage of the device’s maximum power was adopted as a variable. A relatively small spot and a short pulse time of the laser beam made it possible to remelt a small area and cool it quickly. The fast cooling process is beneficial for NiTi alloys, as it preserves the high-temperature B2 phase at room temperature without decomposing into equilibrium phases. In addition, the effect of alloy oxidation is reduced without the need for additional shielding gas. However, the disadvantage of such parameters is the poor mechanical characteristics of the weld. Hence, the formation of the appropriate welding shape (Figure 6b) depended on the laser beam power.

**Figure 7:**
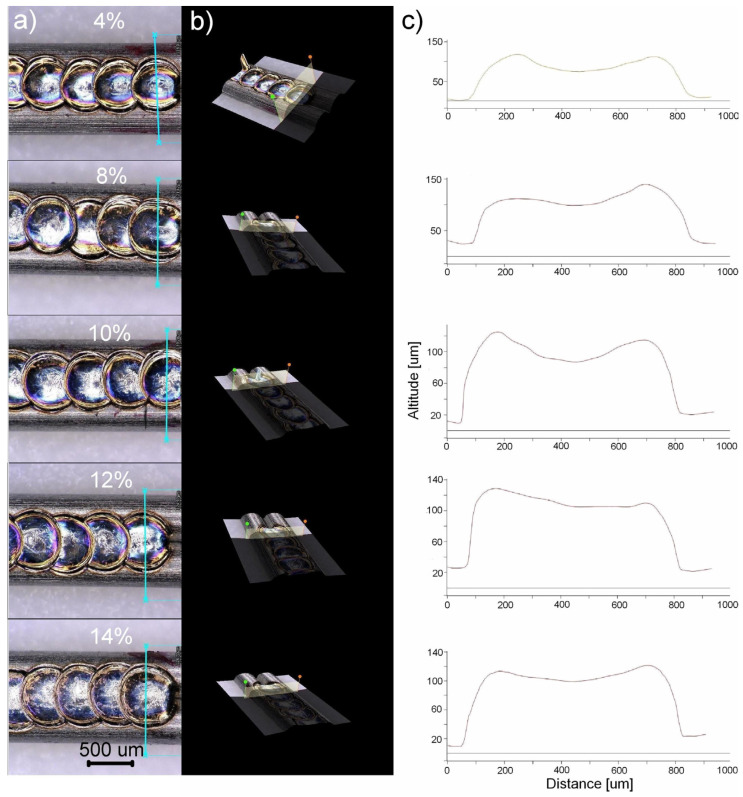
Microscopic image of the weld bead with an example of the weld profile modelling (marked as a blue line) (**a**), the 3D model of the welded area (**b**) and the modelled weld profile (**c**).

Figure 7 shows how the laser power formed the weld bead. Based on the microscopic images, using 3D modelling (Figure 7b), the weld’s cross-section was determined (Figure 7c). The results showed that welding with a laser power lower than 4% produced a small fusion volume (Figure 7—part relating to 4%). On the contrary, the laser power above 16% formed a welding crater. Consequently, the laser power above 50% caused the weld’s gradual, power-dependent perforation. A completely different shape was obtained by applying the 8–14% power. The comparison showed that in that case the fusion profile was the closest to the optimal one (Figure 6b). That is why the welds produced with the 4–14% laser power were selected for further research.

### 3.3. Martensitic Transformation in Welded Wires

**Figure 8:**
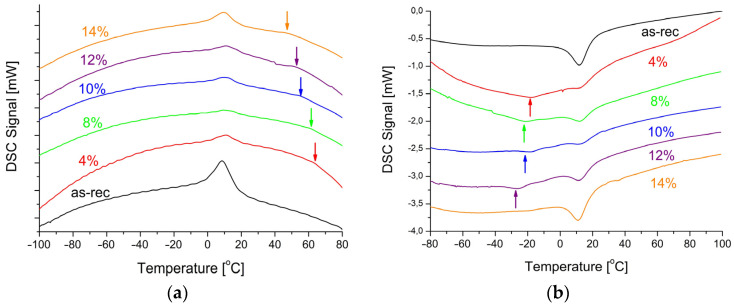
DSC cooling (**a**) and heating (**b**) curves measured for the as-received wire and the welded one with various laser powers.

The reversible martensitic transformation is a measurable effect confirming pseudoelasticity. Its course was examined based on measured thermograms (DSC). The samples containing the area of 2–3 welded points were cut out for the tests. The measured thermograms for the sample in the initial state and the welded wires were summarized in Figure 8. The determined characteristic parameters of martensitic transformation are shown in Table 2.

The occurrence of martensitic transformation and its reversibility is the most important information coming from the thermal analysis. In the DSC cooling curves, there were thermal peaks (Figure 8a) which corresponded to the peaks in the heating direction (Figure 8b). They were broadened, as compared to the ones appearing in the solid material produced via traditional casting. This phenomenon resulted from the grain refinement and/or the increase in the density of structural defects—primarily dislocations. The dislocations are characteristic for the wire production—particularly, while reducing the wire diameter. An additional effect visible on the DSC curves was the multi-stage transformation depending on the applied laser power. The DSC cooling curves showed deviations from the baseline in the 55–75 °C range (indicated by arrows in Figure 8a), and in the −40 °C to −10 °C range in the heating curves (indicated by arrows in Figure 8b). The similar thermal behavior was observed for the laser-treated wire of 100 µm in thickness [27].

Considering the nature of the martensitic transformation, those two facts are not related to each other. Let us first discuss the −40 °C to 10 °C range of heating curves. They may result from the multi-stage martensitic transformation occurring through an additional R-phase which changes the sequence B2↔B19′ to B2↔R↔B19′ [28]. During the cooling process, the B2↔R transformation shows a very narrow temperature range of about 7–10 degrees when the difference between the peak maximum temperatures is measured. This value is characteristic of the B2↔R sequence. In the B2↔B19′ transformation, the necessary difference is 20–30 degrees and it depends on the nickel content. However, in the DSC cooling curves, in the range from −10 °C to practically −100 °C, it was evident that the curves deviated from the usual straight baseline. This may be caused by local stress fields associated with the higher density of dislocations and their clustering under an additional heat treatment, e.g., laser [29]. Consequently, the areas which require cooling undergo a martensitic transformation in different temperature ranges, broadening the thermal peak by up to several dozen degrees. In the reverse transformation, the broad thermal peaks occurred in the range of −40 °C to −10 °C. Increasing the beam power to 14% provided enough energy to rebuild the material’s structure. As a result, the course of the martensitic transformation was similar to the initial wire.

The deviations from the baseline occurring at higher temperatures (55–75 °C) had a completely different character and origin (marked with arrows in Figure 8a). They were not identified as peaks as they did not meet the mathematical criteria adopted to determine the thermal peak. However, it is a fact that the DSC curves change their course in this temperature range. As the delivered heat is proportional to the amount of transforming material, it can be assumed that a small volume in the welded fusion area transforms in this thermal range. Moreover, with the laser power (delivered energy) increase, the main thermal peak enthalpy decreases. This phenomenon is confirmed by a different volume of the material transforming in the discussed temperature range. In order to clarify this aspect, microscopic observations were carried out, and the chemical composition was measured in the weld areas.

**Figure 9:**
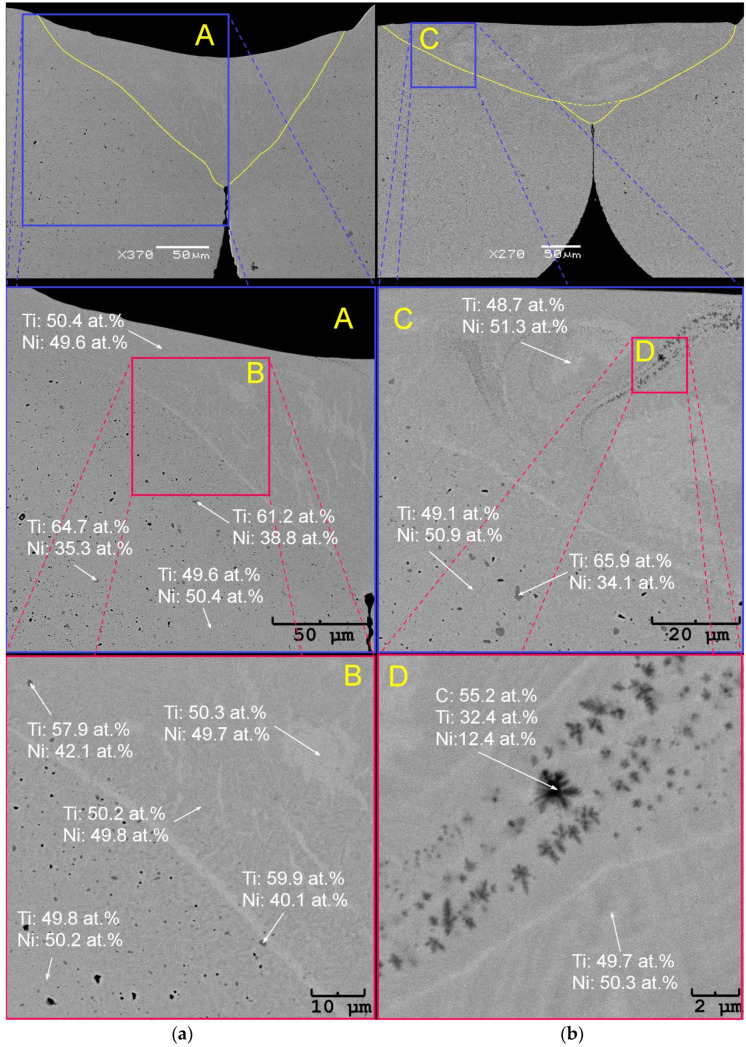
SEM-BS images observed on the transverse cross-section of the wires welded with the power of 4% (**a**) and 14% (**b**).

The observations were performed on the transverse cross-sections of the welded wires. The exemplary images for 4% and 14% power are shown in Figure 9. First, the SEM observations confirmed the 3D modelling from the light microscope images. The SEM images did not reveal the Ti_2_Ni particles in the fusion area, regardless of the applied welding power. At the wire’s initial state, the Ti_2_Ni particles were evenly distributed throughout the entire volume (Figure 3 and Figure 4). In the fusion area, the precipitations completely disappeared. This effect was visible in the enlarged image of the wire welded with the 4% power—Figure 9a—an area marked as “B”. The Ti_2_Ni particles in the fusion zone contained almost 7–10 at.% less titanium than the particles outside the heat-affected zone. In contrast, the particles displayed a slightly bigger amount of nickel as they dissolved in the weld center. However, the particles were depleted in titanium at the periphery of the heat-affected zone. An example is the particle shown in Figure 9a—the enlarged area marked with “A”, located in the heat-affected zone with the titanium content reduced to 61.2 at.%. As titanium passed into the weld area, its amount increased.

The fusion zone contained about 0.4 at.% higher amount of titanium than the rest of the wires. It is a known fact that an increase of about 0.1 at.% Ti raises martensitic transformation temperatures by about 10 °C. In contrast, an increase in the nickel content by 0.1 at.% decreases the transformation temperatures by about 10 °C. The slightly higher titanium content in the fusion zone caused the martensitic transformation. As a result, the broadened maximum appeared in the DSC cooling curves in the 55–75 °C range (Figure 8a). The comparison of the DSC cooling curves indicated the decreased titanium content in the fusion zone, depending on the laser power. The chemical composition of the weld matrix revealed the titanium content lowered to almost 49 at.% (Figure 9b—an area marked as “C”). In addition, the higher the laser power (starting from 8%), the more carbon-containing areas can be found. An example is shown in Figure 9b—the area marked “D”. The inclusion particles contained almost three times more titanium than nickel and up to 55 at.% of carbon. Such inclusions were also observed by the authors [30], identifying them as titanium carbides. Such a distribution of elements proved that the nickel content increase up to 51 at.% caused the martensitic transformation at lower temperatures. It was visible in the DSC heating curves in the −40 °C to −10 °C range. The A_f_ temperature is the most important factor regarding pseudoelasticity. The temperature analysis (Table 2) showed that for the welded wires it was comparable to the temperature of the initial state changing slightly in the 1.5 °C range. Therefore, it can be concluded that pseudoelasticity occurred in the welded wires subjected to an external stress at room temperature.

### 3.4. The Hardness of the Welded Area

**Figure 10:**
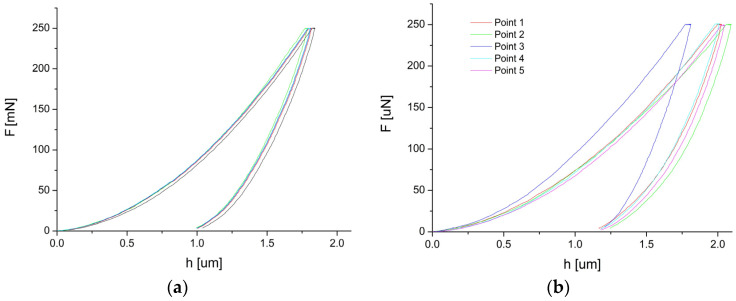
Results of the indentation test done on the wire’s cross-section (**a**) and the weld obtained with the laser power of 10% (**b**).

Microhardness is the material’s resistance to local deformations caused by a mechanical impact on a small area. In the case of the welded wires subjected to an external stress, the microhardness test showed the possible stress concentration areas. The indentation measurements were taken on the wire’s transverse cross-sections and the welded area. The measurements were based on a relationship between the test force applied to the sample—F [mN] and the h [µm] displacement. The results are presented in Figure 10 (the as-received wire—Figure 10a, the 10% laser power sample—Figure 10b). To measure the mechanical properties of the welds, the Vickers hardness HV_IT_ was determined. The values are summarized in Table 3.

The average microhardness determined for the wire before its welding indicated a relatively high value of around 382. These values were characteristic of the B2 parent phase identified in the welded wires. Generally, the B2 phase has a higher hardness than the B19′ martensite. For example, in the laser-treated NiTi alloy which comprised of about 80% B2 phase and 20% B19″ martensite, the micro-hardness ranged from 325 to 340 [31]. Depending on the welding parameters, in the NiTi wire welded to NiTiCu, the microhardness varied from 210 to 260 [32]. Additionally, the hardness value depended on the nickel content in the NiTi alloy. In work [33], the nickel content was 50.7 to 51.27 at.%. The alloy was eviscerated via the laser bed fusion. As a result, the hardness varied from 372 to 724. The chemical composition of the reported wires indicated that the hardness measurements were done for the B2 parent phase. Increasing the nickel content above 50.5 at.% resulted in the Ni_4_Ti_3_ precipitates occurrence, which increased the alloy’s hardness.

**Figure 11:**
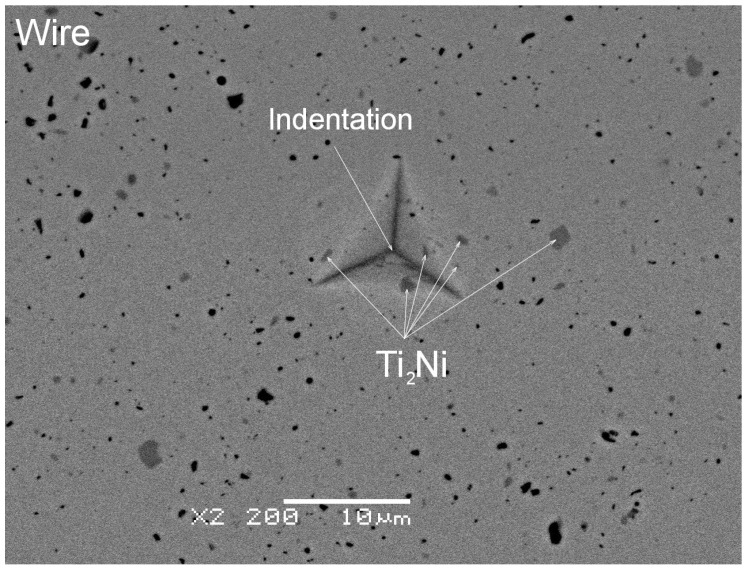
SEM-BS image of the exemplary indentations in the wire.

**Figure 12:**
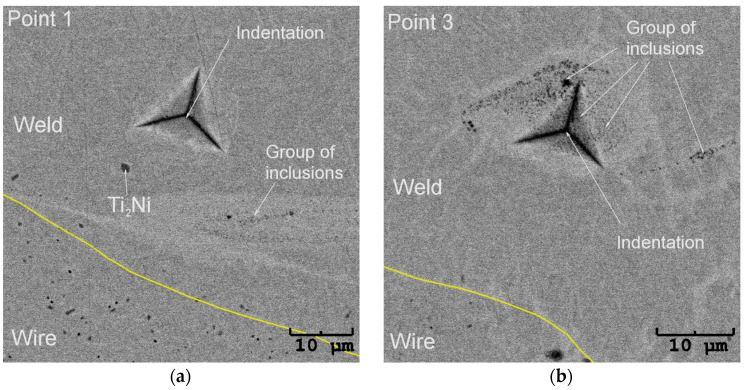
SEM-BS images of the indentations done in the fusion zone for Point 1 (**a**) and Point 3 in the wires welded with the laser power of 10% (**b**).

The determined average microhardness for the wire in the initial state equalled 381, which was slightly higher than the cited literature data. The reason behind this result might be the Ti_2_Ni particles’ presence. Figure 11 shows the SEM image with an exemplary indentation in the wire cross-section. The densely distributed Ti_2_Ni particles can be clearly distinguished from the NiTi matrix. Their influence on the measurement cannot be avoided, as it is known that the increased density of dislocations and the decreasing grain size affect mechanical properties. Such phenomena led to the reduction of the wire diameter and the mechanical properties increase, e.g., hardness. In the fusion area, the Ti_2_Ni precipitates which were characteristic of the wire microstructure in the initial state were not noted. They appeared only sporadically on the edges of the weld. In the heat-affected zone, the chemical composition differed in comparison to the internal part of the wires. The melting process eliminated structural defects such as dislocations. Both facts contributed to the lower microhardness. For the lowest welding power of 4%—the determined hardness decreased to almost 282. However, increasing the welding power caused the microhardness boost, and for 14%, it was 321. This trend was consistent with [31,32]. In our studies, the microhardness increase took place due to inclusions—the (Ti,Ni)C phase containing over 50 at.% of carbon. The particles formed clusters around the remelted weld bead as the laser power increased. For the lower laser powers (e.g., 8%), the clusters were observed only at the surface of the weld face. While increasing the welding power, the clusters appeared throughout the entire welded volume, affecting the microhardness value (Figure 10b). An exemplary microstructure of the indentation area free of inclusions is shown in Figure 12a—marked as Point 1. The representative curve measured for this indent refers to the red line marked in Figure 10b. Its shape and course are characteristic of the remaining measured points, except for Point 3—a blue line in Figure 10b. The microstructure observed for Point 3—Figure 12b—revealed a band of hard inclusions containing the (Ti,Ni)C phase, particularly in the indented area. Consequently, the micro-hardness determined for Point 3 was 360, which was comparable to the wire in the initial state. Increasing the volume fraction of the carbide phase in the weld boosted its hardness.

### 3.5. Pseudoelastic Behaviour of the Welded Wires

**Figure 13:**
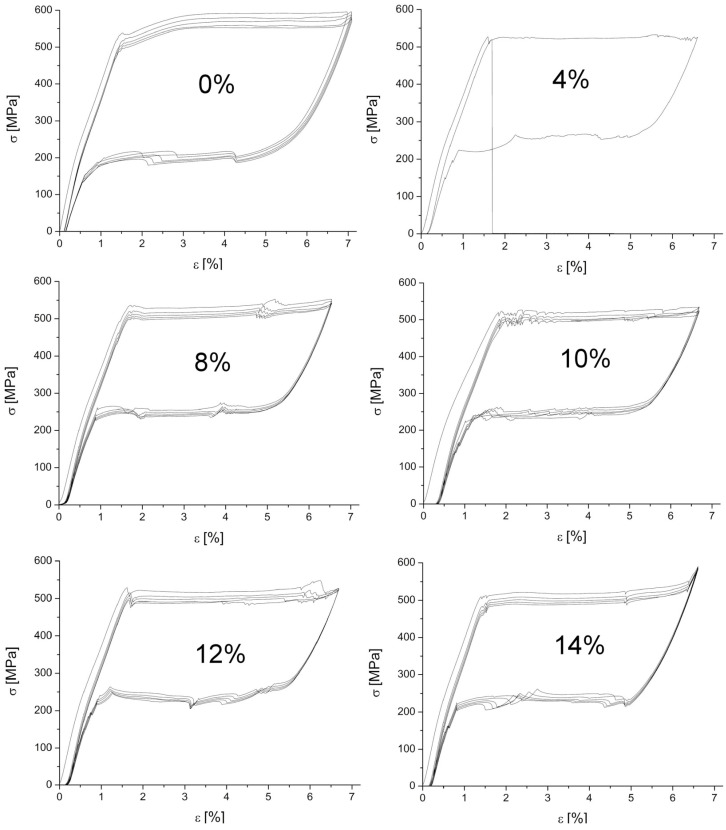
Stress-strain curves measured for the as-received as well as for the welded ones at the various percentages of the maximum (100%) laser beam power.

In order to check pseudoelasticity of the wires, a tensile test was carried out. Preliminary studies showed that the martensitic transformation was completed at a strain lower than 7.2% when the elastic deformation of martensite began. Hence, the experiments were carried out to obtain a maximum elongation of 7% for the as-received wire and 6.8% for the welded wires. The results are summarized in Figure 13. For each sample, five load/unload cycles were measured, except for the wire welded at 4%. After starting the second cycle and reaching the critical stress to trigger the martensitic transformation, the sample crashed, which proved the fusion zone’s amount, size, and/or shape to be inadequate. The stresses concentrated at the boundary of the heat-affected zone and the parent wires were high enough to destroy the joint. After the first cycle, all the samples behaved similarly and the residual plastic deformation appeared, as reported in the literature [23]. The loops were closed in the remaining cycles, regardless of the applied welding power.

**Figure 14:**
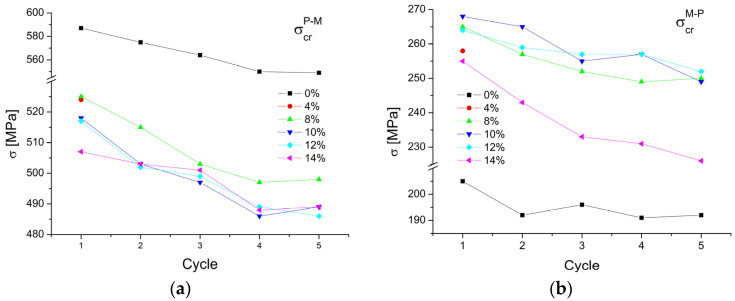
The critical stress triggering the martensitic transformation for the forward (**a**) and reverse course (**b**).

The critical stress to induce the martensitic transformation was calculated from the load/unload curves. The results, depending on the applied laser power, are shown in Figure 14. In the case of the forward martensitic transformation, the critical stress reached the highest value of 587 MPa for the as-received wire. Similar results were obtained by the authors in [20]. The cyclic load/unload repetition decreased the critical stress to 549 MPa for the fourth cycle, and for the fifth cycle it remained the same. This means that after four cycles, the martensitic transformation began to stabilize. The samples welded with the 4% and 8% powers revealed a similar strain of 524 MPa. The 10% and 12% welded samples required 518 MPa to induce martensite. Welding with the 14% power significantly reduced the critical stress value to 507 MPa, which was notably different from the wire’s initial state. The fourth and fifth cycle load/unload gave similar results to the initial state results, regardless of the applied welding power.

In the case of the reverse transformation, for the as-received wire, the stress inducing the martensitic transformation was the lowest and amounted to about 190 MPa. For the welded wires (the laser power from 4% to 12%), the first cycle required about 265 MPa to start the reverse transformation. For 14%, it was the lowest one—255 MPa. This means that for the welded specimens, the wire began to return to its original shape at a higher stress.

Summing up the critical stresses required to initiate the martensitic transformation, the value decreased by about 6% after five cycles, as compared to the first cycle. This may result from structural changes in the welded area and the heat-affected zone. The spreading heat acted locally as an annealing factor, decreasing the material’s density and/or clustering structural defects. It is known that heat affects the wire structure reconstruction and the grain growth after the production stage deformation.

## 4. Conclusions

The studies carried out on the welded wires, obtained with a simple laser apparatus and a variable power parameter, revealed the valid dependency. The following conclusions can be drawn from the research results:

- The applied laser beam power influenced the shape of the weld. The power ranged from 12% to 14%, assuming the complete filling of the space between the welded wires was achieved. However, the laser power above 14% caused the crater formation and the perforation of the joint.
- The fusion zone received with the low laser powers (below 8%) was free of Ti_2_Ni precipitations, present in the entire volume of the wire, except for the weld. However, increasing the laser power caused turbulences of the liquid weld material, accommodating inclusions of nickel titanium carbide into the weld.
- The weld microhardness was characteristic of the B2 parent phase and increased with the higher laser power from 282 (4%) to 321 (14%). This tendency resulted from the inclusions as the laser power increased.
- Welding affected the martensitic transformation course by lowering its enthalpy and extending the temperature range it occurred in.
- The higher laser power lowered the critical stress needed to induce pseudoelasticity—from 570 MPa for the initial state of the wire to 507 MPa for the wire welded with the 14% power.
- For the wire in the initial state and the welded samples—regardless of the applied welding power—the reversible martensitic transformation course stabilized after four load/unload cycles. Moreover, as pseudoelasticity was cyclic, the critical stress was reduced by 6–7%.

## Figures and Tables

**Table 1:** Average chemical composition (at.%) determined at the wires’ cross-section.

Alloying Element	Perpendicular	Longitudinal
Ti	49.7 ± 0.4	49.7 ± 0.4
Ni	50.3 ± 0.4	50.3 ± 0.4

**Table 2:** Transformation temperatures and enthalpies determined for the as-received wires as well as the welded one.

Power [%]	M_s_ [°C]	M_f_ [°C]	ΔH [J/g]	A_s_ [°C]	A_f_ [°C]	ΔH [J/g]
As-received	18.9	−4.9	3.39	0.3	20.6	2.85
4%	17.3	−1.8	0.9	−33.3	21.9	2.9
8%	22.3	0.2	1.84	−31.4	22.6	2.04
10%	21.6	1.6	1.58	−27.3	22.8	1.55
12%	20.3	−1.1	1.87	−36.9	21.01	2.07
14%	20.3	1.3	3.11	1.02	19.94	2.48

**Table 3:** Average Vickers hardness in dependency on the applied laser power.

Laser Power [%]	Vickers Hardness *HV_IT_*	Remarks
0	381.9 ± 10.2	wire
4	282.4 ± 13.8	weld
8	302.1 ± 21.1	weld
10	304.6 ± 28.2	weld
12	310.8 ± 20.6	weld
14	321.4 ± 26.4	weld

## Data Availability

The data presented in this study are available on request from the corresponding authors.
